# Hypertensive crisis in patients with obstructive sleep apnea-induced hypertension

**DOI:** 10.1186/s12872-021-02119-x

**Published:** 2021-06-23

**Authors:** Sittichai Khamsai, Apichart Chootrakool, Panita Limpawattana, Jarin Chindaprasirt, Wattana Sukeepaisarnjaroen, Verajit Chotmongkol, Songkwan Silaruks, Vichai Senthong, Yuwares Sittichanbuncha, Bundit Sawunyavisuth, Kittisak Sawanyawisuth

**Affiliations:** 1 Department of Medicine, Faculty of Medicine, Khon Kaen University, 123 Mitraparp Road, Khon Kaen, 40002 Thailand; 2 Department of Emergency Medicine, Mahidol University, Ramathibodi Hospital, Bangkok, Thailand; 3 Department of Marketing, Faculty of Business Administration and Accountancy, Khon Kaen University, Khon Kaen, Thailand

**Keywords:** Systolic blood pressure, Obstructive sleep apnea, Hypertensive urgency, Hypertensive emergency

## Abstract

**Background:**

Hypertensive crisis is an urgent/emergency condition. Although obstructive sleep apnea (OSA) in resistant hypertension has been thoroughly examined, information regarding the risk factors and prevalence of hypertensive crisis in co-existing OSA and hypertension is limited. This study thus aimed to determine prevalence of and risk factors for hypertensive crisis in patients with hypertension caused by OSA.

**Methods:**

The inclusion criteria were age of 18 years or over and diagnosis of co-existing OSA and hypertension. Those patients with other causes of secondary hypertension were excluded. Patients were categorized by occurrence of hypertensive crisis. Factors associated with hypertensive crisis were calculated using multivariate logistic regression analysis.

**Results:**

There were 121 patients met the study criteria. Of those, 19 patients (15.70%) had history of hypertensive crisis. Those patients in hypertensive crisis group had significant higher systolic and diastolic blood pressure at regular follow-ups than those without hypertensive crisis patients (177 vs. 141 mmHg and 108 vs. 85 mmHg; *p* value < 0.001 for both factors). After adjusted for age, sex, and Mallampati classification, only systolic blood pressure was independently associated with hypertensive crisis with adjusted odds ratio (95% CI) of 1.046 (1.012, 1.080).

**Conclusions:**

The prevalence of hypertensive crisis in co-existing OSA and hypertension was 15.70% and high systolic blood pressure or uncontrolled blood pressure associated with hypertensive crisis in patients with OSA-associated hypertension.

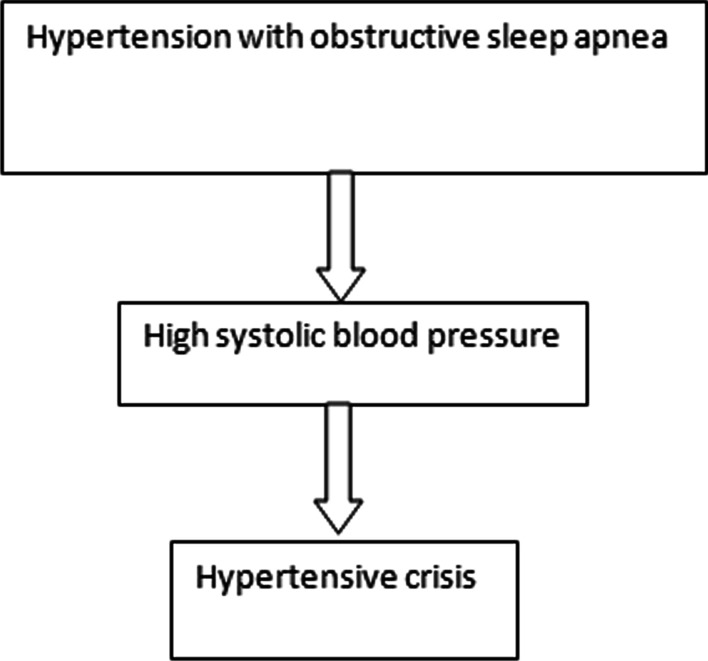

## Introduction

Obstructive sleep apnea (OSA) is a disease that is commonly encountered in clinical practice. Its estimated prevalence is approximately 26% in the population between 30 and 70 years of age [1]. It causes intermittent desaturations during sleep, which can result in various cardiovascular diseases such as hypertension, heart failure, atrial fibrillation, coronary artery disease, and stroke [2].

In 2003, OSA was found to be a common cause of hypertension [3]. The prevalence of OSA in hypertensive patients is approximately 50% and ranges from 30–80% [4]. The prevalence of OSA in patients with resistant hypertension is up to 71% which was similar to that in those with hypertensive crisis [5–7]. Large neck circumference, snoring, and age are predictors of OSA in these patients with adjusted odds ratios of 4.7 (1.3–16.9), 3.7 (1.3–11.0), and 5.2 (1.9–14.2), respectively [6]. However, although OSA in resistant hypertension has been thoroughly examined, information regarding the risk factors and prevalence of hypertensive crisis in co-existing OSA and hypertension is limited. This study thus aimed to determine prevalence of and risk factors for hypertensive crisis in patients with hypertension caused by OSA.

## Methods

This was a retrospective study conducted at Khon Kaen University Hospital Hypertension clinic in Thailand. The inclusion criteria were age of 18 years or over and diagnosis of co-existing OSA and hypertension. The diagnosis of hypertension was based on the criteria proposed in the JNC 7 [3], while that of OSA was made according to the apnea-hyponea index (AHI; five or more apnea or hypopnea events per hour). Although hypertension can have various causes, this study examined only patients with hypertension caused by OSA and excluded all others. The study period was between 2015 and 2016.

We collected clinical data from the medical charts of all eligible patients recorded at the last follow-up including clinical features, symptoms and signs of OSA, co-morbid diseases, cardiovascular diseases, and laboratory investigation results. The outcome of this study was presence of hypertensive crisis at the Emergency Department, which was diagnosed as systolic and/or diastolic blood pressure greater than 180/110 mmHg [8]. Cases in which there was acute target organ damage from hypertensive crisis, such as heart failure or papilledema, were defined as hypertensive emergency, while those in which there was no acute target organ damage from hypertensive crisis were recorded as hypertensive urgency.

Sample size calculation. A previous report found the prevalence of hypertensive crisis in the Emergency Department to be 11.5% [9]. Based on a formula for a single population, we determined that the expected prevalence of hypertensive crisis in OSA patients was likely to be 20%. Thus, the required sample size was 77 to reach the expected OSA prevalence of 20% in hypertensive crisis with a confidence of 90% and power of 80%.

All eligible patients were classified by the presence of hypertensive crisis. Clinical factors of patients in both groups were compared using descriptive statistics. Factors associated with hypertensive crisis were calculated using logistic regression analysis. Univariate logistic regression analysis was used to identify the risk factors for hypertensive crisis. Those with *p* values less than 0.20 or clinically significant were included in the subsequent multivariate logistic regression analysis. Results of the logistic regression analyses were presented as unadjusted and adjusted odds ratio (OR) with 95% confidence interval (CI). All analyses were performed using STATA version 10.1 (College Station, Texas, USA).

## Results

During the study period, there were 121 patients with hypertension caused by OSA. Of those, 19 (15.70%) had a history of hypertensive crisis, categorized as either hypertensive urgency (15 patients) or hypertensive emergency (four patients). There were two significant factors that differed between patients with and without history of hypertensive crisis (Table 1): systolic and diastolic blood pressure. Those with a history of hypertensive crisis had higher median systolic and diastolic blood pressure than those without (systolic: 177 vs. 141 mmHg; diastolic: 108 vs. 85 mmHg). Symptoms and signs of OSA, co-morbid diseases, and cardiovascular diseases were comparable between the two groups.

**Table 1:** Clinical features of patients with hypertension secondary to obstructive sleep apnea (OSA) categorized by presence of hypertensive crisis (HTC)

Factors	No HTC n = 102	HTC n = 19	*p* value
*Basic characteristics*			
Age, years	50.5 (39.0–59.0)	42.0 (33.0–52.0)	0.062
Male sex	56 (54.90)	9 (47.37)	0.620
BMI, kg/mm^2^	29.3 (26.0–35.6)	30.6 (26.3–34.8)	0.917
SBP, mmHg	141 (130–150)	177 (150–190)	< 0.001
DBP, mmHg	85 (79–93)	108 (90–121)	< 0.001
Previous alcohol consumption*	14 (31.82)	3 (30.00)	0.999
Current alcohol consumption	9 (20.45)	1 (10)	0.667
Previous smoker*	7 (15.91)	2 (20.00)	0.667
Current smoker	3 (6.98)	0 (0.00)	0.999
Wearing dentures	3 (5.56)	0 (0.00)	0.999
No. of antihypertensive drugs	1 (1–2)	2 (1–3)	0.081
Statin therapy	56 (60.22)	11 (61.11)	0.999
*Signs and symptoms of OSA*			
Snoring	61 (98.39)	12 (92.31)	0.319
Median snoring duration, years	8.0 (3.5–10.0)	2.0 (2.0–20)	0.500
Witnessed apnea	26 (70.27)	5 (83.33)	0.659
Nocturia, times/night	2 (1–3)	2.5 (1–4)	0.439
Morning headache	19 (50.00)	3 (75.00)	0.608
Unrefreshing sleep	26 (78.79)	5 (71.43)	0.645
Excessive daytime sleepiness	47 (87.04)	6 (75)	0.328
Mallampati classification			0.092
@1	0 (0)	(7.69)	
@2	22 (35.48)	(23.08)	
@3	29 (46.77)	(46.15)	
@4	11 (17.74)	2 (15–38)	
Macroglossia	30 (81.08)	6 (66.67)	0.384
Torus palatinus	8 (32.00)	2 (28.57)	0.999
Torus mandibularis	6 (25.00)	1 (14.29)	0.999
Tonsil enlargement (%)	7 (21.88)	1 (14–29)	0.999
Retrognathia (%)	5 (22.73)	2 (28.57)	0.999
Median neck circumference, cm	41.0 (38.0–44.7)	37.0 (35.0–38.0)	0.089
*Co-morbid diseases*			
Diabetes mellitus	33 (35.48)	4 (26.67)	0.572
GERD	32 (39.51)	6 (40.00)	0.999
Allergic rhinitis	20 (50.00)	3 (33.33)	0.472
*Cardiovascular events*			
Stroke	8 (8.99)	1 (7.69)	0.999
Coronary artery disease	8 (8.89)	2 (14.29)	0.621
Heart failure	9 (10.00)	2 (13.33)	0.656
Atrial fibrillation	2 (2.22)	1 (7.14)	0.355
Other arrhythmias	2 (2.25)	1 (7.69)	0.339

Data recorded at the last follow-up at the clinic prior to HTC occurrence for HTC group and last follow-up at the clinic during study period for the no HTC group; data presented as numbers (percentage) or median (1st to 3rd quartile range)

*BMI* body mass index, *SBP* systolic blood pressure, *DBP* diastolic blood pressure, *GERD* gastroesophageal reflux disease

^*^Indicated no alcohol consumption or smoking at all after cessation

There was no significant difference between the two groups in terms of laboratory results (Table 2). The hypertensive crisis group had a lower average apnea hypopnea index score than those without hypertensive crisis (14.5 vs. 19.5 events/hour; *p* value 0.363). After adjusting for age, sex, and Mallampati classification, only systolic blood pressure was independently associated with hypertensive crisis, with an adjusted odds ratio (95% CI) of 1.046 (1.012, 1.080).

**Table 2:** Laboratory results of patients with hypertension secondary to obstructive sleep apnea categorized by presence of hypertensive crisis (HTC)

Factors	No HTC n = 102	HTC n = 19	*p* value
Polysomnography			
@AHI, events/hr	19.5 (10.0–35.0)	14.5 (5.0–29.0)	0.363
@Lowest oxygen saturation (%)	81.5 (72.0–88.0)	83 (72.0–89.0)	0.712
BUN, mg/dL	12.3 (9.2–16.0)	12.5 (11.1–15.7)	0.549
Cr, mg/dL	0.9 (0.7–1.1)	0.9 (0.7–1.3)	0.920
ALT, U/L	30 (21–55)	19 (14–43)	0.179
AST, U/L	26 (20–42)	27 (15–53)	0.749
HbA1c, %	6.2 (5.7–7.3)	5.8 (5.4–6.3)	0.143
UACR, mg/d	12 (5–53)	403 (39–657)	0.157
Cholesterol, mg/dL	186 (167–212)	170 (143–237)	0.392
Triglyceride, mg/dL	120 (101–168)	111 (88–163)	0.508
HDL, mg/dL	46 (40–57)	46 (40–53)	0.540
LDL, mg/dL	118 (104–150)	115 (85–175)	0.966

Data presented as median (1st to 3rd quartile range)

*AHI* apnea–hypopnea index, *BUN* blood urea nitrogen, *Cr* creatinine, *ALT* alanine aminotransferase, *AST* aspartate aminotransferase, *UACR* urine albumin-creatinine ratio, *HDL* high density lipoprotein, *LDL* low density lipoprotein, *RVSP* right ventricular systolic pressure, *NA* not applicable

## Discussion

Hypertensive crisis (particularly hypertensive emergency) has been associated with high morbidity and mortality. A French study reported 15 deaths out of 46 patients (33%) within three months after admission with hypertensive emergency [10]. Previous studies have found the general prevalence of hypertensive crisis in hypertensive patients to be approximately 1–2%, of which 25% presented with hypertensive emergency [11, 12]. The population in this study was patients with hypertension caused by OSA. The prevalence of hypertensive crisis in this setting was much higher than in the general population (15.70% vs. 1–2%), but the proportion of patients with hypertensive emergency was comparable between this study and general population (21.1% vs. 25%) [11]. These results may indicate that patients with hypertension caused by OSA are at higher risk for hypertensive crisis. A previous study also confirmed this with evidence that OSA was a cause of hypertensive crisis in 70% of the161 patients examined [7].

One previous study in 89 hypertensive patients found female sex and obesity to be significantly associated with hypertensive crisis [13]. Our study found that in patients with co-existing OSA and hypertension, baseline systolic blood pressure was a significant risk factor for hypertensive crisis (Table 3). The median systolic blood pressure of the hypertensive crisis group was significantly higher than that of the non-hypertensive crisis group (177 vs. 141 mmHg), as shown in Table 1. We found similar results with regard to diastolic blood pressure. These findings were similar to those of a previous report from the US [14], which found that uncontrolled systolic blood pressure in an out-patient setting increased the risk of hypertensive crisis by 1.30 times. Note that the study population in the that report was not limited to OSA patients, as it was in this study.

**Table 3:** Factors associated with occurrence of hypertensive crisis in patients with hypertension secondary to obstructive sleep apnea

Factors	Unadjusted odds ratio (95% confidence interval)	Adjusted odds ratio (95% confidence interval)
Age, years	0.970 (0.936, 1.005)	0.996 (0.947, 1.048)
Male sex	1.353 (0.507, 3.609)	0.908 (0.205, 4.021)
Systolic blood pressure, mmHg	1.074 (1.041, 1.108)	1.046 (1.012, 1.080)
Mallampati classification	1.076 (0.879, 1.317)	1.051 (0.905, 1.222)

Other OSA risk factors, such as age over 50 years, large neck circumference, and snoring, have been found to be significant predictors for OSA in cases of resistant hypertension (odds ratios of 5.2, 4.7, and 3.7, respectively) [6]. Our study found, however, that the correlations between these factors and hypertensive crisis in patients with hypertension caused by OSA were not statistically significant (Tables 1 and 3). These findings may be explained by the fact that this study only included patients with OSA, while the previous study included patients with all types of secondary hypertension.

There were some limitations in this study. First, the criteria for diagnosis of hypertensive crisis varies across studies [15]. In this study, we used those laid out in the GEAR project [8, 10, 16, 17], while some other studies have used diagnostic criteria such as systolic/diastolic blood pressure of 200/110 mmHg [3, 15]. However, had we used the latter definition, it would not have affected patient enrollment in this study. Numbers of eligible patients for both criteria were similar. Second, no causes/outcomes of hypertensive crisis or symptoms/signs preceding crisis were studied due to the retrospective study design. Therefore, OSA cannot be assumed to be the cause of hypertensive crisis. However, our aim was to determine the risk factors for hypertensive crisis in these patients, not to show a causal relationship between OSA and hypertensive crisis. Note that DBP was not included in the final model as it has collinearity with SBP, and there were few co-morbidities in the study population. Finally, this study was limited in that other aspects of OSA were not studied [18–20].

## Conclusion

The prevalence of hypertensive crisis in patients with co-existing OSA and hypertension was 15.70%. High systolic blood pressure or uncontrolled blood pressure associated with hypertensive crisis in patients with OSA-associated hypertension.

## Data Availability

All relevant data are available from the corresponding author on reasonable request.
